# Treatments for gastrointestinal symptoms in acute food protein-induced enterocolitis syndrome

**DOI:** 10.5415/apallergy.0000000000000185

**Published:** 2025-02-05

**Authors:** Masaaki Hamada, Yoshihiko Sakurai, Ichiro Tanaka

**Affiliations:** 1 Department of Paediatrics, Yao Municipal Hospital, Osaka, Japan; 2 Department of Legal Medicine, Nara Medical University, Kashihara, Japan

**Keywords:** 5-HT_3_ receptor inhibitors, food protein-induced enterocolitis syndrome, granisetron, guideline, hydrocortisone

## Abstract

The treatment for acute food protein-induced enterocolitis syndrome (FPIES) has not been fully resolved. We investigated the efficacy of 5-HT_3_ receptor inhibitors and steroids in patients with recurrent vomiting during the oral food challenge test (OFC) for acute FPIES. Patients who met the diagnostic criteria of the international guidelines of acute FPIES and developed recurrent vomiting with the OFC were enrolled. Patients who had a single vomiting underwent infusion with normal saline. A 5-HT_3_ receptor inhibitor was administered intravenously when recurrent vomiting appeared with lethargy and/or pallor. If the patient continued to develop persistent symptoms, additional steroids were administered intravenously. This study examined the rate of OFCs in which the administration of a single 5HT_3_ inhibitor was insufficient to improve gastrointestinal symptoms, requiring the additional administration of steroids. A total of 20 OFCs were included; 2 treated with infusion therapy, 9 OFCs treated with 5-HT_3_ receptor inhibitor, and 9 OFCs treated with 5-HT_3_ receptor inhibitors and steroids. Nine OFCs treated with a single 5-HT_3_ receptor inhibitor were effective, with only 2 episodes of vomiting observed before administration, and none after treatment. In the remaining 9 OFCs, steroids were additionally administered due to persistent symptoms. Five of 7 OFCs with persistent vomiting improved, and all 7 OFCs with persistent lethargy improved. This study revealed that in OFCs performed due to acute FPIES leading to recurrent vomiting, monotherapy with 5-HT_3_ receptor inhibitors was insufficient in half of the OFCs, suggesting that combination therapy with 5-HT_3_ receptor inhibitors and steroids should be considered for severe OFCs.

## 1. Introduction

Food protein-induced enterocolitis syndrome (FPIES) is a non-IgE-mediated food allergy characterized by delayed and potentially severe gastrointestinal symptoms in the absence of cutaneous and respiratory symptoms [1].

In the first international consensus guidelines for acute FPIES, the treatment methods for gastrointestinal symptoms have been presented to clinicians [1]. The initial treatment for mild to moderate gastrointestinal symptoms, mainly vomiting, is oral rehydration and a subsequent administration of ondansetron, a 5-HT_3_ receptor inhibitor. In patients with severe symptoms, methylprednisolone is deemed the next step for treatment [1], although the mechanism of its effectiveness and the optimal timing for its administration remains unclear, thus requiring further investigation.

In acute FPIES, 5-HT_3_ receptor inhibitors are known to be effective and more effective than methylprednisolone against gastrointestinal symptoms [2, 3], but the additional treatment for patients in which insufficient efficacy is noted and symptoms other than vomiting are present after treatment remains unclear. Few studies have examined the efficacy of 5-HT_3_ receptor inhibitors and the rate of additional treatment for the gastrointestinal symptoms of acute FPIES, prompting us to investigate these aspects.

## 2. Methods

### 2.1. Study design

This single-center, hospital-based, retrospective case series was designed to evaluate 5-HT_3_ receptor inhibitors and steroids.

### 2.2. Patients

We recruited patients who met the diagnostic criteria of the international guidelines for acute FPIES and developed recurrent vomiting during the oral food challenge test (OFC) from 2021 to 2023 [1]. We analyzed the patients’ background factors (age at onset, allergenic food, clinical history, total IgE titer, specific IgE antibody titer to allergic foods, and comorbid allergic disease) at the first OFC. The specific IgE levels were measured using Alastat 3 g Allergy (Siemens Healthcare Japan, Osaka, Japan).

### 2.3. OFC

OFC was conducted with a single ingestion under direct medical supervision, and the patients were observed for >4 hours after ingestion. The challenge dose was set at 2 g for any patient in each OFC (eg, 2 g egg yolk, 2 g yogurt, or 2 g udon noodles).

Patients who had vomited at least once underwent infusion with normal saline. Granisetron (0.04 mg/kg), a 5-HT_3_ receptor inhibitor available at our hospital, was administered intravenously under electrocardiographic monitoring when recurrent vomiting appeared with lethargy and/or pallor. If the patient continued to develop vomiting, lethargy, or pallor for >30 minutes after granisetron administration, additional hydrocortisone (5 mg/kg) was administered intravenously. Hydrocortisone was used because of its immediate effect and short elimination half-life. When symptoms improved after single granisetron administration at a given OFC, that OFC was considered a part of the monotherapy group; when hydrocortisone was added to granisetron, that OFC was considered a part of the combination group.

The primary outcome was the rate of OFCs in which the administration of a single 5HT_3_ inhibitor was insufficient to improve gastrointestinal symptoms, requiring the additional administration of steroids.

### 2.4. Statistical analysis

We used the median and interquartile ranges (IQRs) due to variables with skewed distributions. The Mann–Whitney *U* and Fisher exact tests were used to compare the medians and proportions. Statistical analyses were performed using EZR version 4.2.2 (Saitama Medical Center, Jichi Medical University, Saitama, Japan), which is a graphical user interface for R (The R Foundation for Statistical Computing, Vienna, Austria) [4] and *P <* 0.05 was deemed statistically significant.

### 2.5. Ethics

This study was approved by the Ethics Committee of Yao Municipal Hospital, Osaka, Japan (Chairperson Dr T. Morimoto) on May 1, 2023 (approval number: YMH-010523-184). Written informed consent was obtained from the parents or guardians of all patients.

## 3. Results

**Figure 1.**
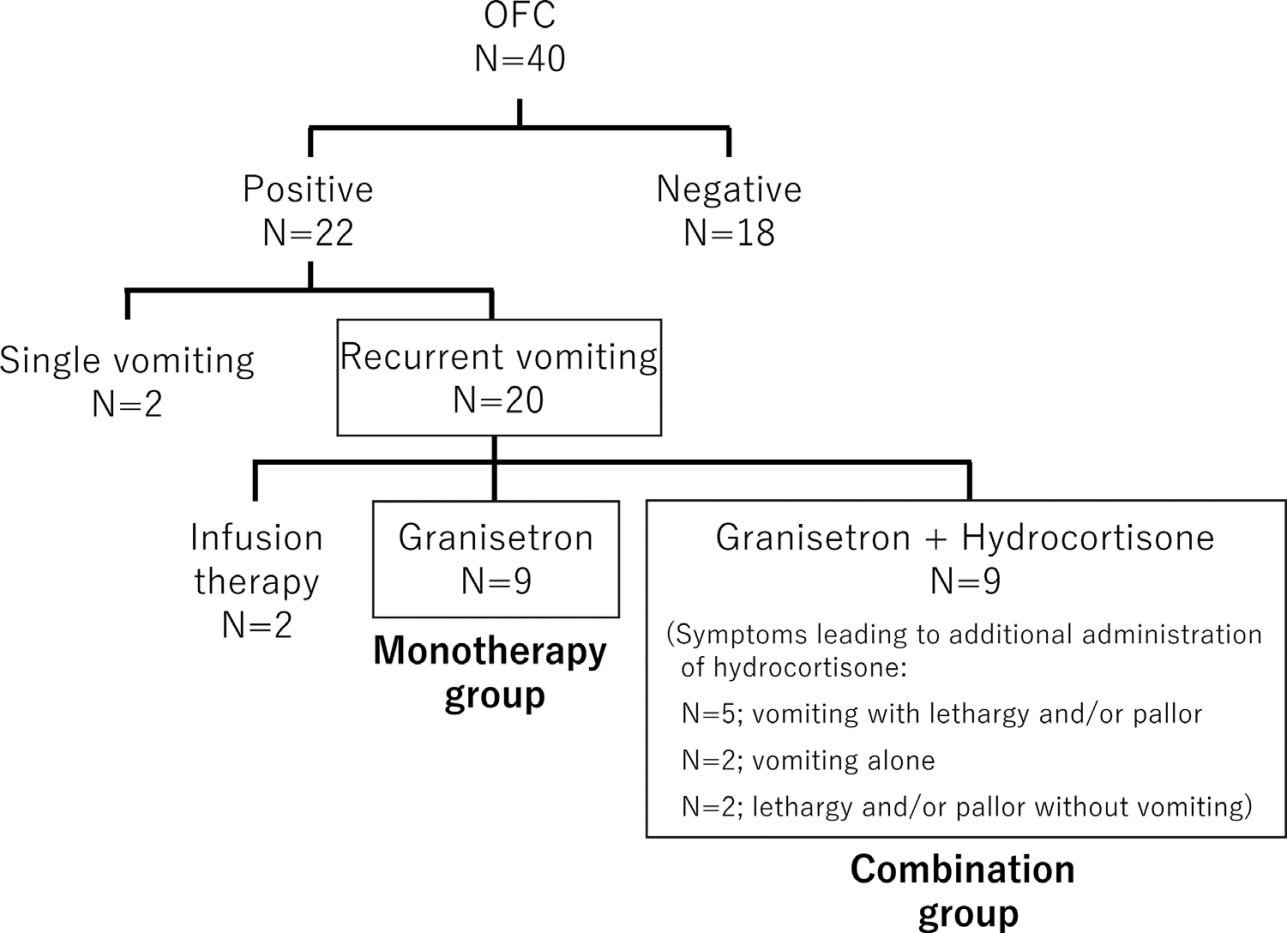
Study flowchart showing the distribution based on OFC results. Treatment comprised infusion therapy, hydrocortisone, and granisetron. Patients who received granisetron alone were assigned to the monotherapy group, while those who received both granisetron and hydrocortisone were assigned to the combination group. OFC, oral food challenge test; N, number of OFCs.

During the observation period, 40 OFCs were performed in 25 patients from 10 to 24 months of age. Among 22 positive OFCs, a single vomiting episode was observed in 2 OFCs, and recurrent vomiting occurred in 20 OFCs (17 with egg yolk, 2 with wheat, and 1 with milk) in 15 patients (Fig. 1 and Table 1). Of the 20 OFCs that were included in this study, since each patient in 2 OFCs had 2 vomiting episodes without lethargy or pallor, they only received infusion therapy. Granisetron was administered in 18 OFCs. No electrocardiographic abnormalities were observed during or after administration in all patients receiving granisetron.

**Table 1.** Characteristics of patients with recurrent vomiting in OFC

	Patients
Number of patients	15
Age at onset, median (IQR), mo	8.0 (7–9)
Male, n (%)	4 (26.7)
Allergenic foods	
Egg yolk, n (%)	13 (86.6)
Milk, n (%)	1 (6.7)
Wheat, n (%)	1 (6.7)
Major criterion based on clinical history, n (%)	15 (100.0)
Minor criteria based on clinical history	
A second (or more) episode of repetitive vomiting, n (%)	15 (100.0)
Repetitive vomiting episode after eating different foods, n (%)	1 (6.7)
Extreme lethargy, n (%)	12 (80.0)
Marked pallor, n (%)	14 (93.3)
Need for emergency department visit, n (%)	3 (21.4)
Need for intravenous fluid support, n (%)	1 (6.7)
Diarrhea in 24 h (usually 5–10 h), n (%)	0 (0.0)
Hypotension, n (%)	0 (0.0)
Hypothermia, n (%)	0 (0.0)
Total IgE titer, median (IQR), IU/mL	16.0 (6.5–45.0)
Specific IgE antibody titer to allergic foods, median (IQR), IUA/mL	0.29 (0.16–0.81)
IgE-mediated food allergy, n (%)	1 (6.7)
Comorbid disease	
Atopic dermatitis, n (%)	6 (40.0)
Bronchial asthma, n (%)	0 (0.0)
Allergic rhinitis, n (%)	0 (0.0)

Major and minor criteria were based on international consensus guidelines [1].

FPIES, food protein-induced enterocolitis syndrome; IQR, interquartile range; OFC, oral food challenge test.

In the monotherapy group, comprising 9 OFCs in which granisetron was effective, only 2 episodes of vomiting were observed before administration, and none occurred after administration. In the remaining 9 OFCs, hydrocortisone was additionally administered due to persistent symptoms: vomiting, lethargy, and/or pallor complexion in a total of 5 OFCs, vomiting alone in 2 OFCs, and lethargy and/or pallor in 2 OFCs. The monotherapy and combination groups comprised 9 OFCs each (Fig. 1 and Table 2).

**Table 2.** Results of OFC in monotherapy and combination groups

	Monotherapy group	Combination group	*P* value
Number of OFCs	9	9	
Age at OFC, median (IQR), mo	12 (10–24)	16 (16–20)	0.478
Allergenic foods			
Egg yolk, n (%)	8 (88.9)	7 (77.8)	1.000
Milk, n (%)	1 (11.1)	0 (0.0)	1.000
Wheat, n (%)	0 (0.0)	2 (22.2)	0.471
Overnight hospitalization, n (%)	0 (0.0)	3 (33.3)	0.206
Time to onset of symptoms, median (IQR), min	180 (120–180)	120 (120–150)	0.313
Time to improvement of symptoms, median (IQR), min	240 (210–240)	360 (300–360)	0.024
Time from granisetron administration to resolution of vomiting, median (IQR), min	0 (0–0)*	75 (60–120)	<0.001
Total number of vomiting episodes, average ± SD	2.0 ± 0.0†	3.1 ± 0.9	0.002
Major criterion based on OFC, n (%)	9 (100.0)	9 (100.0)	1.000
Minor criteria based on OFC			
Vomiting, n (%)	9 (100.0)	9 (100.0)	1.000
Lethargy, n (%)	9 (100.0)	9 (100.0)	1.000
Pallor, n (%)	5 (55.6)	6 (66.7)	0.107
Diarrhea 0–5 h after food ingestion, n (%)	0 (0.0)	0 (0.0)	1.000
Hypotension, n (%)	0 (0.0)	0 (0.0)	1.000
Hypothermia, n (%)	0 (0.0)	0 (0.0)	1.000
Neutrophil count increase of more than 1,500 from baseline‡, n (%)	2 (22.2)	5 (55.6)	Not tested
Symptoms before hydrocortisone use			
Vomiting with lethargy/pallor, n (%)		5 (55.6)	
Vomiting only, n (%)		2 (22.2)	
Lethargy/pallor without vomiting, n (%)		2 (22.2)	

Major and minor criteria were based on international consensus guidelines [1].

IQR, interquartile range; OFC, oral food challenge test; SD, standard deviation.

* No vomiting after granisetron administration.

† In the monotherapy group, vomiting occurred twice in all OFCs.

‡ Comparisons with baseline were made in 7 OFCs.

The time to the onset of symptoms was not different in the monotherapy and the combination group. Overnight hospitalization was not required for any OFCs in the monotherapy group, whereas it was required for 3 out of 9 OFCs in the combination therapy group, with no significant difference observed between the groups (Table 2).

In the combination group, the median time to resolution of vomiting was 75 minutes (IQR, 60–120 minutes) after granisetron administration; the total number of vomiting episodes was 3.1 ± 0.9. In the combination group, a total of 7 OFCs experienced recurrent vomiting after granisetron administration, which was resolved after hydrocortisone administration in 5 OFCs (71.4%). Of the remaining 2 OFCs with persistent vomiting after hydrocortisone administration, one developed vomiting 30 minutes after administration but no further emesis occurred; one other patient developed vomiting 1 hour after administration; the patient initially improved, but vomiting returned the next morning and improved after the administration of granisetron and hydrocortisone. All 7 OFCs with persistent lethargy and/or pallor improved after the administration of hydrocortisone (Table 2).

## 4. Discussion

Our results showed that 50% of patients with acute FPIES treated with granisetron for recurrent vomiting upon OFC had fully improved, while the remaining 50% had inadequate improvement and required hydrocortisone administration. This suggests that granisetron alone may not be sufficient in some patients to fully improve gastrointestinal symptoms in acute FPIES.

In acute FPIES, the differentiation in administration between 5-HT_3_ receptor inhibitors and steroids for gastrointestinal symptoms has not been clarified. Miceli Sopo et al. investigated drug effects on emetic symptoms undergoing OFC in 51 patients with acute FPIES: 37 received ondansetron and 14 received methylprednisolone. In the ondansetron group, vomiting improved in 30/37 patients (81%), significantly higher than the proportion (1/14 patients; 7%) in the methylprednisolone group [3]. The present study showed a higher occurrence of persistent vomiting after the administration of granisetron compared with that in a previous report [3]. Granisetron and ondansetron reportedly have equal efficacy for postoperative vomiting [5]. The efficacy of granisetron and ondansetron varies in reported studies, with different reports suggesting different levels of superiority [6, 7]. It has been reported that in pediatric patients (4–11 years) with acute lymphocytic leukemia, 4 mg of ondansetron and 1 mg of granisetron are effective for vomiting, and granisetron has a more significant antiemetic effect [6]. In contrast, a meta-analysis of the efficacy of 5-HT_3_ receptor inhibitors in gastroenteritis reported that only ondansetron was effective [7]. Whether granisetron and ondansetron have different effects on emesis during OFC and whether the dose of granisetron was sufficient to warrant further study.

Although the exact mechanism of the antiemetic effect of steroids is unclear, previous studies suggest several mechanisms. The combination of a 5-HT_3_ receptor inhibitor and a steroid in chemotherapy reduces vomiting more effectively than a 5-HT_3_ receptor inhibitor alone [8]. Steroids and 5HT_3_ receptor inhibitors act in an additive manner, each acting on the 5-HT_3_ receptor [9]. Furthermore, steroids suppress and inhibit serotonin secretion [10] and exert an antiemetic effect by acting on the central nervous system [11].

In acute FPIES, improvement of vomiting as the primary symptom is important, but attention should also be paid to patients whose lethargy and/or pallor persists even after improvement in vomiting. In the combination group, adding hydrocortisone improved persistent lethargy and/or pallor that did not respond adequately to granisetron also improved. OFCs involving persistent lethargy and/or pallor may be considered for the additional use of steroids after the administration of 5-HT_3_ receptor inhibitors.

Although symptoms may differ between adult and pediatric patients with acute FPIES, 9 of 22 patients (40.9%) with recurrent vomiting in adult acute FPIES reported having a fear of death, requiring an appropriate response to the provoking symptoms [12]. It is sometimes difficult to assess the child’s anxiety, and both the child and the parents may be hesitant to proceed with the next OFC due to the fear of repeated vomiting. If 5-HT_3_ receptor inhibitors and steroids are administered according to the symptoms with OFC for acute FPIES, patients may recover without trauma.

The limitations of this study were its retrospective design and the absence of a control group, making it impossible to determine whether there was an effect of additional steroid administration or the natural course, which treatment—5-HT_3_ receptor inhibitors or steroids—would be more appropriate for OFCs with persistent vomiting, and a risk factor for severe OFCs, along with a limited number of patients.

In conclusion, 5-HT_3_ receptor inhibitors were not fully effective in treating recurrent vomiting with lethargy and/or pallor in acute FPIES during half of the OFCs, suggesting that additional treatment should be necessary in severe OFCs, and steroids may be a potential option for adjunctive therapy.

## Acknowledgements

The authors would like to thank all patients who participated in this study and their parents.

## Conflicts of interest

The authors have no financial conflicts of interest.

## Author contributions

MH designed the study and wrote the initial draft of the manuscript. IT and YS assisted in the preparation of the manuscript and critically reviewed the manuscript. All authors have read and approved the final manuscript.
